# Hypoxia and Hypoxia Mimetics Decrease Aquaporin 5 (AQP5) Expression through Both Hypoxia Inducible Factor-1α and Proteasome-Mediated Pathways

**DOI:** 10.1371/journal.pone.0057541

**Published:** 2013-03-01

**Authors:** Jitesh D. Kawedia, Fan Yang, Maureen A. Sartor, David Gozal, Maria Czyzyk-Krzeska, Anil G. Menon

**Affiliations:** 1 Department of Molecular Genetics, Biochemistry and Microbiology, University of Cincinnati, Cincinnati, Ohio, United States of America; 2 Department of Biostatistics, University of Michigan, Ann Harbor, Michigan, United States of America; 3 University of Chicago Comer Children’s Hospital, Chicago, Illinois, United States of America; 4 Department of Cancer and Cell Biology University of Cincinnati, Cincinnati, Ohio, United States of America; 5 VA Research Service, Department of Veterans Affairs, Cincinnati, Ohio, United States of America; 6 Department of Molecular Medicine, City of Hope Comprehensive Cancer Center, Duarte, California, United States of America; 7 Department of Pharmacy Research, University of Texas MD Anderson Cancer Center, Houston, Texas, United States of America; New York Medical College, United States of America

## Abstract

The alveolar epithelium plays a central role in gas exchange and fluid transport, and is therefore critical for normal lung function. Since the bulk of water flux across this epithelium depends on the membrane water channel Aquaporin 5 (AQP5), we asked whether hypoxia had any effect on AQP5 expression. We show that hypoxia causes a significant (70%) decrease in AQP5 expression in the lungs of mice exposed to hypoxia. Hypoxia and the hypoxia mimetic, cobalt, also caused similar decreases in AQP5 mRNA and protein expression in the mouse lung epithelial cell line MLE-12. The action of hypoxia and cobalt on AQP5 transcription was demonstrated by directly quantifying heternonuclear RNA by real-time PCR. Dominant negative mutants of Hypoxia Inducible Factor (HIF-1α) and HIF-1α siRNA blocked the action of cobalt, showing that HIF-1α is a key component in this mechanism. The proteasome inhibitors, lactacystin or proteasome inhibitor-III completely abolished the effect of hypoxia and cobalt both at the protein and mRNA level indicating that the proteasome pathway is probably involved not only for the stability of HIF-1α protein, but for the stability of unidentified transcription factors that regulate AQP5 transcription. These studies reveal a potentially important physiological mechanism linking hypoxic stress and membrane water channels.

## Introduction

Aquaporins are a family of membrane water channels that are required for the transport of water through many secretory and absorptive epithelia [1], [2], [3]. Aquaporin 5 (AQP5), a member of the AQP family is highly expressed in the mammalian lung, brain, salivary glands, and lachrymal glands. In the lung, it is expressed on the apical surface of both type I and type II alveolar epithelial cells [4], [5]. Although it is known that a significant decrease in airway-capillary water permeability is seen in the lungs of mice in which AQP5 is deleted [6], acute lung injury does not appear to affect AQP5 deficient mice differently from their wild-type counterparts, prompting the question of what role AQP5 may play in the mammalian lung [7].

Hypoxic stress occurs in many physiologic and pathologic conditions, such as decrease in alveolar oxygen tension during ascent to high altitude, or as a consequence of hypoventilation related to central nervous disorders, obstructive airway disease, or acute lung injury [8], [9]. Previous studies have shown that hypoxia and Co^++^ affect the expression of a number of genes that play a central role in remodeling the lung in response to hypoxic stress, including up-regulation of the transcriptional activator hypoxia-inducible factor (HIF-1α) [10], [11], [12], sometimes considered a “master regulator” of adaptive responses to hypoxia.

Since it is well known that the alveolar epithelium in the lung is a key anatomical site for both gas exchange and fluid transport, we considered the possibility that oxygen tension regulates the expression of AQP5, and tested this hypothesis by examining the effect of hypoxic stress on AQP5 expression in lungs of mice exposed to hypoxia and in the mouse lung epithelial cell line MLE-12. We established the experimental conditions for hypoxic stress initially using both hypoxic chambers (1% oxygen for 24 h) and by adding cobalt chloride (Co^++^), a well-established hypoxia mimetic [13], [14], [15], [16]. Once the system was calibrated, addition of Co^++^ was used as the inducer of hypoxic stress, based on ease of use.

Here we show that hypoxia and the hypoxia mimetic cobalt significantly decrease AQP5 expression at both the mRNA and protein levels in the MLE-12 lung epithelial cell line, and HIF-1α and proteasomes are the key molecular components of the signaling system involved in the transduction of the hypoxic stress signal to AQP5. These findings reveal a potentially important physiological link between hypoxic conditions in the cell and the expression of AQP5, and contribute to our understanding of disorders of fluid handling in the lung.

## Results

### Exposure to the Hypoxia Mimetic, Cobalt, Results in Decreased Expression of Both AQP5 Protein and mRNA

**Figure 1:**
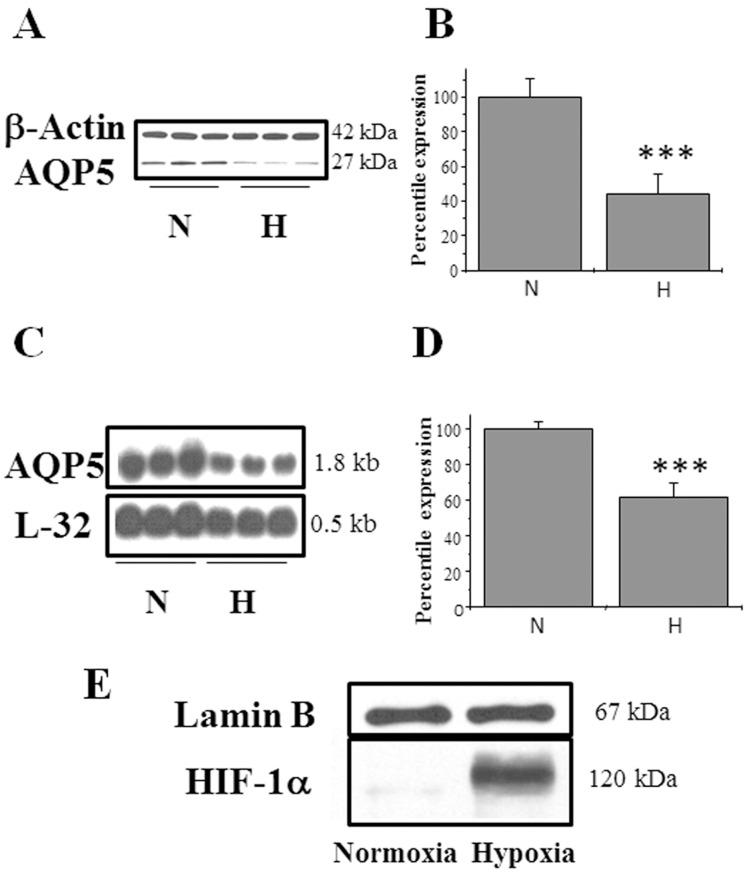
Hypoxia decreases expression of AQP5 protein and mRNA levels in MLE-12 cells. (A) Western blot analysis of total protein extract from MLE-12 cells exposed to normoxia and hypoxia (B) Quantitation of the Western blots using β-actin as the loading control (C) Northern blot analysis of total mRNA isolated from cells exposed to normoxia and hypoxia. (D) Quantitation of the Northern blots blot using L32 as the loading control. (E) Western blot analysis of nuclear extracts from cells exposed to normoxia and hypoxia. Lamin B was used as a loading control. Values for the blots are the mean ± SEM (n = 3). N, normoxia. H, hypoxia.

**Figure 2:**
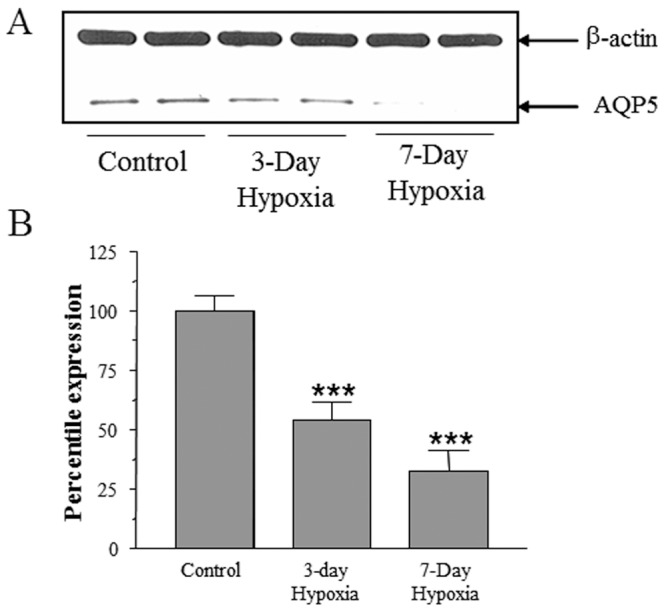
AQP5 expression is decreased in lung of mice exposed to hypoxia. (A) Western blot analysis of total protein extracts isolated from lungs of mice exposed to hypoxia and controls. (B) Quantitation of the Western blots using β-actin as the loading control. Values in the plot are the mean ± SEM (controls-n = 5; 3 day hypoxia-n = 6; 7 day hypoxia-n = 7, p<0.0001 using ANOVA).

To investigate whether hypoxia affects AQP5 expression, MLE-12 cells were exposed to 1% O_2_ for 24 h in a hypoxic chamber, and total RNA or protein extracts were prepared after addition of a chaotropic agent that prevented reoxygenation. Western blot analyses of total protein extracts showed a 60% decrease in AQP5 protein levels compared to normoxic controls (Fig. 1A and B). Northern blot analyses of total RNA showed that expression of AQP5 mRNA levels decreased by 40% when compared with normoxic controls (Fig. 1C and D). Levels of HIF-1α protein, which is known to be increased in response to hypoxia, were measured (Fig. 1E) as a positive control for hypoxic stress in the cells. The physiological relevance *in vivo* was confirmed when AQP5 protein were decreased by 50% in mice exposed 8% to hypoxia for 3 days and by almost 70% in mice exposed to hypoxia for 7 days in hypoxic chamber (Fig. 2).

**Figure 3:**
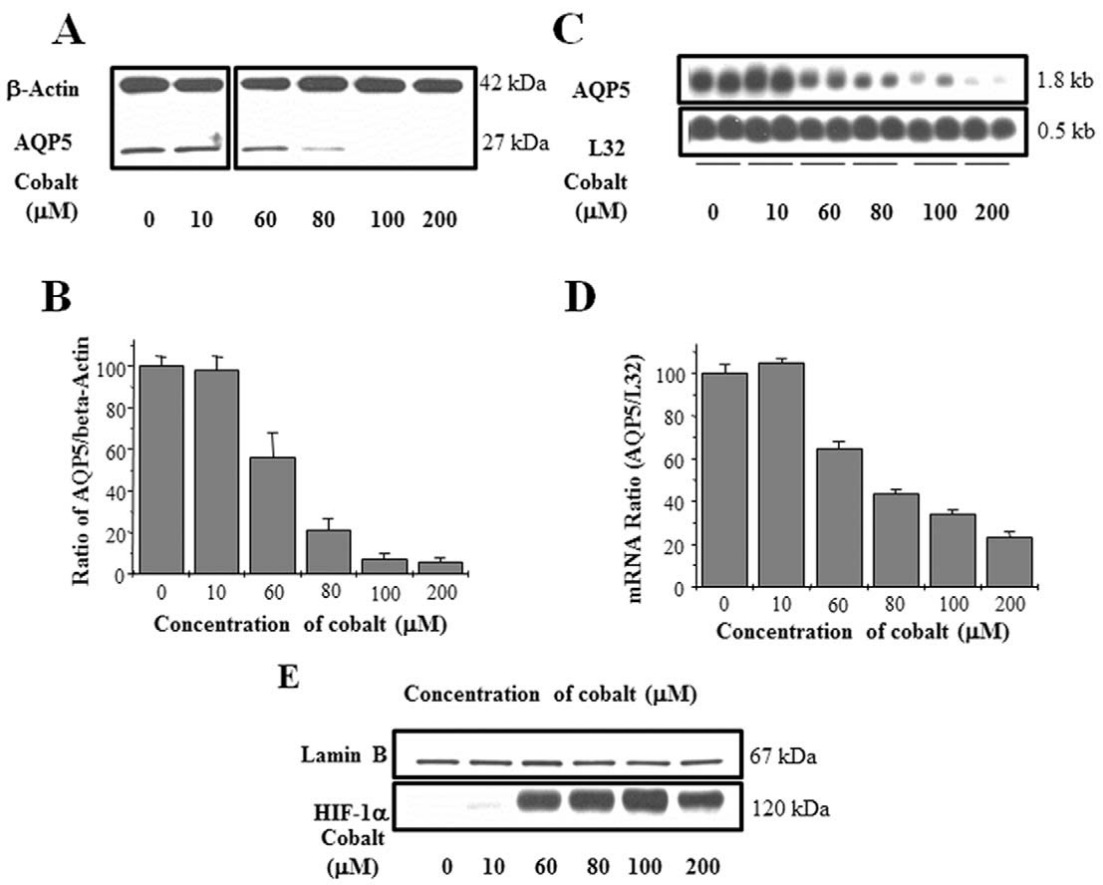
Cobalt decreases the expression of AQP5 expression in MLE-12 cells. (A) Western blot analysis of total protein extracts isolated from MLE-12 cells incubated in media containing 0, 10, 60, 80, 100, or 200 μM cobalt for 24 h respectively. (C) Northern blot analysis of total mRNA isolated from cells treated with 0, 10, 60, 80, 100, or 200 μM Cobalt for 24 h respectively. (B) Quantitation of the Western blots using β-actin as the loading control. (D) Quantitation of the Northern blots blot using L32 as the loading control. (E) Western blot analysis of nuclear extracts from cells exposed to cobalt. Lamin B was used as a loading control. Values in the blot are the mean ± SEM (n = 3).

To determine whether the hypoxia mimetic cobalt could mimic the effect of hypoxia on AQP5 at both the mRNA and protein levels, MLE-12 cells were incubated in media containing 10, 60, 80, 100, or 200 µM Co^++^ for 24 h before the isolation of total protein or total RNA. Western blot (Fig. 3A and 3B) and Northern blot analyses (Fig. 3C and D) showed that AQP5 expression decreased by Co^++^ in a dose-dependent manner. Treatment with Co^++^ caused a greater decrease in AQP5 expression at the protein level (90%) when compared to the decrease in AQP5 mRNA (60% at 100 µM, and 75% at 200 µM dose of Co^++^). Cobalt also increased protein expression of HIF-1α, (which served as a positive control for cellular hypoxia) in a dose-dependent manner as shown in Fig. 3E. Since the maximal effect on AQP5 protein could be attained with 100 µM Co^++^, this concentration was used for all subsequent experiments.

### Exposure to Cobalt Results in Decreased AQP5 Transcription

Decrease in AQP5 mRNA expression could be due to decrease in mRNA stability or decrease in AQP5 transcription. Cobalt treatment did not increase the degradation of AQP5 mRNA. If anything, a slight but statistically insignificant increase in the stability of AQP5 mRNA was observed after cobalt treatment (data not shown).

**Figure 4:**
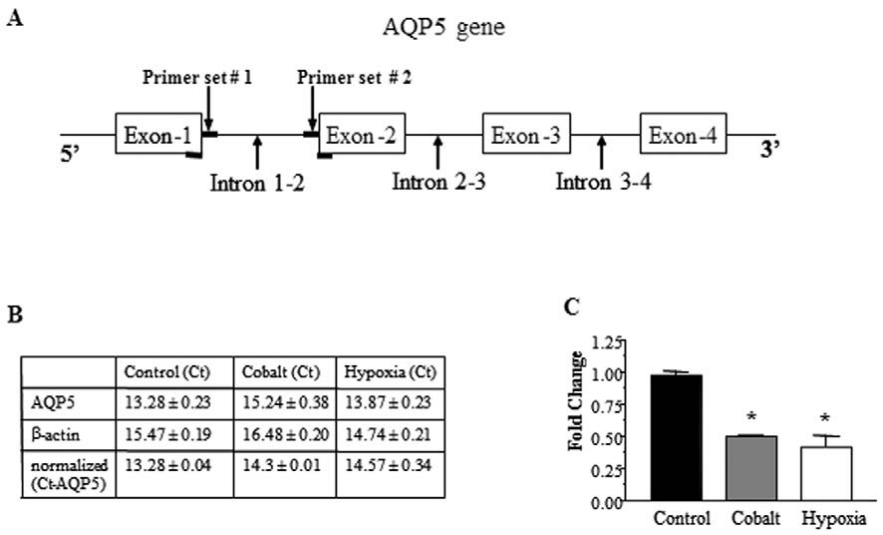
Hypoxia and Cobalt decrease AQP5 mRNA by down-regulating AQP5 transcription. (A) Schematic representation of the location of primers used to selectively amplify AQP5 hetero nuclear RNA (hnRNA) from control and hypoxic stressed cells. (B) Ct values of AQP5 and β-actin from real time PCR. (C) Quantitation of the AQP5 hnRNA in panel B using normalized β-actin expression levels (n = 3).

Direct measurement of AQP5 transcription was performed by quantifying heteronuclear RNA (hnRNA), in which total RNA was prepared from untreated, hypoxia and cobalt treated cells. After DNAse treatment to remove potential residual genomic DNA contamination, cDNA was prepared and amplified by Real Time PCR using two independent sets of primers designed to selectively amplify intron- exon fragments (Fig. 4 A thus amplifying cDNA that represented heteronuclear RNA (hnRNA) and not amplifying mRNA). The results, shown in Fig. 4B and 4C, show a twofold decrease in AQP5 transcription.

### The AQP5 Response to Cobalt Requires De Novo Protein Synthesis

**Figure 5:**
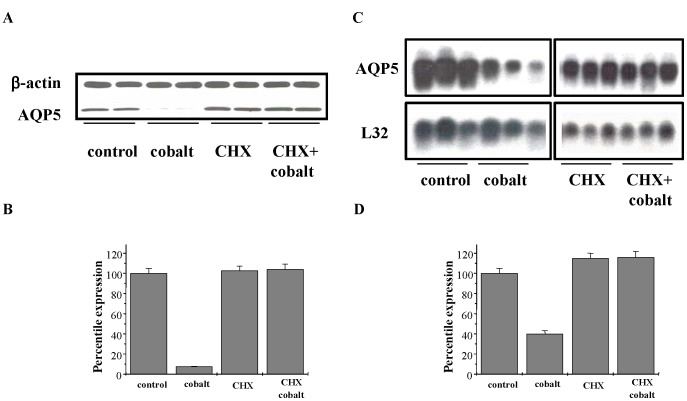
*de novo* protein synthesis is necessary to down-regulate AQP5 expression. (A) Western blot analysis of MLE-12 cells pre-incubated with cycloheximide or vehicle (70% ethanol) followed by 100 μM cobalt. (B) Quantitation of the Western blots in panel B using β-actin as the loading control. (C) Northern blot analysis of MLE-12 cells pre-incubated with cycloheximide or vehicle (70% ethanol) followed by 100 μM cobalt. (D) Quantitation of the Northern blots in panel E using L32 as the loading control. Values in the all the blots are the mean ± SD (n = 3).

To examine whether *de novo* protein synthesis was required for cobalt-induced decrease in AQP5 mRNA and protein, MLE-12 cells were pretreated with cycloheximide (CHX) (500 µg/ml) for 30 min, a dose previously shown to significantly block protein synthesis [17]. Subsequent addition of 100 µM cobalt for 24 h, followed by the isolation of total RNA and total protein for analysis by Northern blot and Western blot as shown in (Fig. 5). Preincubation with CHX completely abolished the effect of Co^++^ on AQP5 protein, but CHX alone did not affect levels of the AQP5 protein (Fig. 5 A–B). Similarly, preincubation with CHX completely blocked the effect of Co^++^ on AQP5 mRNA but CHX alone did not alter AQP5 mRNA (Fig. 5 C and D).

### HIF-1α is Necessary for the Regulation of AQP5 Expression Under Hypoxic Conditions

**Figure 6:**
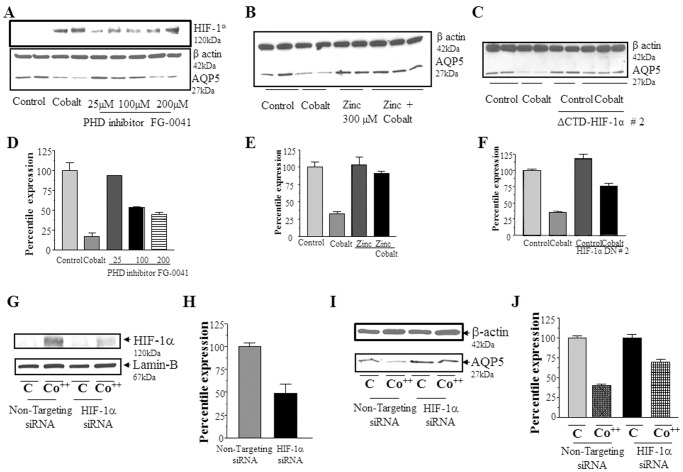
HIF-1α is necessary to regulate AQP5 expression under hypoxic conditions. (A) Western blot analysis to determine AQP5 expression (bottom panel) in total protein extracts isolated from cells treated with vehicle, cobalt, PHD inhibitor-0041 at 25 µM, 100 µM, and 200 µM conc. Similarly, expression of HIF-1α in the top panel and expression of β-actin in the middle panel in the same groups. (B) Western blot analysis to determine AQP5 expression in total protein extracts isolated from cells treated with vehicle, cobalt, zinc chloride, cobalt plus zinc chloride (bottom panel) and β-actin (top panel). (C) Western blot analysis to determine AQP5 expression (bottom panel) and β-actin (top panel) in total protein extracts isolated from mock transfected MLE-12 cells, and MLE-12 cells transfected with HIF-1α dominant negative, treated with vehicle and cobalt. (D) Quantitation of the AQP5 expression using β-actin as the loading control in panel A. (E) Quantitation of AQP5 expression using β-actin as the loading control in panel B. (F) Quantitation of the AQP5 expression using β-actin as the loading control in panel C. (G) Western blot analysis to determine expression of HIF-1α (top panel) and Lamin B (bottom panel) in total protein extracts isolated from cells transfected with non-targeting siRNA and HIF-1α siRNA. Transfected cells were treated with vehicle and cobalt. (H) Quantitation of the HIF-1α expression using Lamin B as the loading control in panel G. (I) Western blot analysis to determine expression of AQP5 (bottom panel) and β-actin (top panel) in total protein extracts isolated from cells transfected with non-targeting siRNA and HIF-1α siRNA. Transfected cells were treated with vehicle and cobalt. (J) Quantitation of the AQP5 expression using β-actin as the loading control in panel I. Values for the blots are the mean ± SD (n = 3).

Multiple approaches were used to determine whether HIF-1α is necessary to regulate AQP5 expression when cells were treated with hypoxia mimetic cobalt. It has been previously shown that inhibiting prolyl hydroxylase (PHD) enzyme blocks the hydroxylation of HIF-1α, thereby preventing the proteasome-mediated degradation of HIF-1α. Addition of PHD-inhibitor-FG-0041 resulted in decreased expression of AQP5 (Fig. 6A, lower panel) in a dose-dependent manner with the maximum effect (50% decrease) observed at 200 µM (Fig. 6D). Thus the level of AQP5 protein is inversely correlated to the level of HIF-1α protein. (Fig. 6A, top and lower panels). FG-0041 was used because it has been shown to be the most effective among multiple PHD inhibitors that were tested [18] and has also been shown to be more selective in its inhibition of PHD enzymes than other commonly used inhibitors such as dimethyloxaloylglycine [19].

It has also been shown that zinc chloride triggers the inhibition of HIF-1α by inducing an endogenous, alternative spliced variant, which does not have the nuclear translocation signal, but can bind to ARNT and sequester that in the cytosol [20]. The overall effect is that this HIF-1α variant acts as a dominant negative of HIF-1α [20]. Pre-treatment of MLE-12 cells with zinc chloride (300 µM) completely blocked the effect of hypoxic stress but had no effect on AQP5 expression in the absence of hypoxic stress (Fig. 6B and 6E), providing further evidence that high levels of HIF-1α are correlated with low levels of AQP5.

A major potential drawback of pharmacological inhibitors is the difficulty in evaluating their specificity. We therefore, transfected MLE-12 cells with dominant negative mutants of HIF-1α, selected stable cell lines that over-expressed this mutant, and measured the levels of AQP5 in these cell lines with or without exposure to Co^++^_._ Exposure of cells expressing the dominant negative HIF-1α to Co^++^ resulted in a significant inhibition in decrease in AQP5 protein levels (25%) compared to a, 75% decrease in mock transfected controls, showing that blocking the action of the endogenous HIF-1α using the dominant negative mutant directly rescued AQP5 protein levels (Fig. 6C and F).

To eliminate the possibility that selecting dominant negative HIF-1α stable cell lines from parental MLE-12 cells could result in artifacts, we directly tested the hypothesis that decreasing HIF-1α would rescue the expression of AQP5 after Co^++^ treatment by transfecting MLE-12 cells with siRNA designed to block expression of HIF-1α. Transfection with siRNA targeted to HIF-1α resulted in a 50% decrease in expression of HIF-1α protein when compared to the non-targeting, negative control siRNA (Fig. 6G and 6H). When the same Western blot was probed for AQP5, knockdown of HIF-1α resulted in a 2-fold rescue of the action of Co^++^ (Fig. 6I and 6J).

### The Effect of Cobalt on AQP5 Expression is Mediated Through the ERK Signaling Pathway

**Figure 7:**
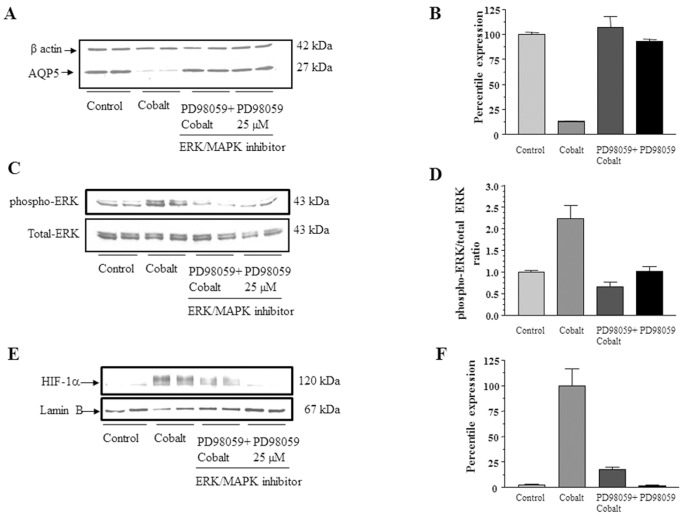
Cobalt regulates AQP5 expression via ERK signaling pathway. (A) Western blot analysis to determine AQP5 expression in total protein extracts isolated from cells treated with vehicle, cobalt, cobalt plus ERK pathway inhibitor PD98059 and PD98059 alone. (B) Quantitation of the Western blots in panel A using β-actin as the loading control. (C) Western blot analysis to determine phosphorylated ERK expression (upper panel) in total protein extracts isolated from cells shown in panel A. Total ERK expression in the same groups (lower panel). (D) Quantitation of the change in phosphorylation of ERK using total ERK for normalization. (E) Western blot analysis to determine HIF-1α expression in nuclear protein extracts isolated from cells treated with vehicle, cobalt, cobalt plus ERK pathway inhibitor PD98059 and PD98059 alone. (F) Quantitation of the Western blots in panel E using Lamin B as the loading control. Values for the blots are the mean ± SD (n = 3).

To investigate whether cobalt decreases AQP5 expression via the ERK signaling pathway, cells were treated with PD98059 (ERK pathway inhibitor) prior to treatment with Co^++^, and the effect of inhibitor was evaluated by measuring the rescue of AQP5 expression. Treatment of cells with PD98059 blocked the effect of Co^++^ on the expression of AQP5 as shown in Fig. 7 A and B. Hypoxic stress increased the phosphorylated form of ERK by 2-fold, which could be completely blocked by PD98059 (Fig. 7 C and D), and the inhibitor alone had no measurable effect on the phosphorylated form of ERK. Blocking the ERK pathway also inhibited the action of hypoxic stress on HIF-1α (Fig. 7E), showing that the hypoxic stress signal is processed by the ERK pathway upstream of HIF-1α.

### Proteasome Inhibitors Abolish the Effect of Cobalt on AQP5 Protein and mRNA Expression

**Figure 8:**
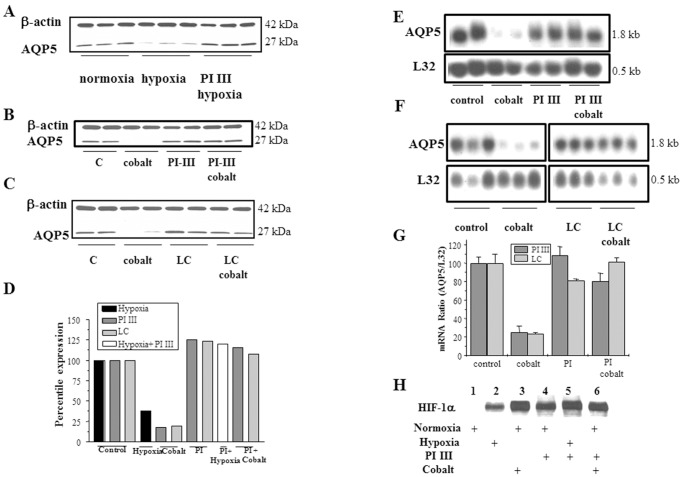
Hypoxia and Cobalt regulate expression of AQP5 via a proteasome dependent pathway. (A) Western blot analysis to determine AQP5 expression in total protein extracts isolated from cells exposed to normoxia, hypoxia or hypoxia with 10 μm proteasome inhibitor PI III for 24 h. β-actin is used as a loading control. (B)& (C) Western blot analysis of total protein extracts of cells were pre-treated with 10 μM proteasome inhibitor PI III or LC for 30 min, and then 100 μM Cobalt was added for 24 h. β-actin is used as loading control. (D) Quantitation of Western blots in panels A–C. (E)& (F) Northern blot analysis of total mRNA isolated from cells, which were pre-treated with 10 μM proteasome inhibitor PI III or LC for 30 min, and then 100 μM cobalt was added for 24 h. L32 is used as a loading control. (G) Quantitation of Northern blots in panels E and F. (H) Western blot analysis to determine HIF-1α expression in nuclear extracts from differently treated cells. Lane 1, normoxia; lane 2, hypoxia; lane 3, normoxia plus cobalt; lane 4, normoxia plus PI III; lane 5, hypoxia plus PI III; lane 6, normoxia plus PI III and cobalt. Values for the blots are the mean ± SD (n = 3).

Microarray analysis of MLE-12 cells treated with vehicle or Co^++^ showed that expression of alpha type-5, 7; beta type-4,10; 26 ATPase-1; and 26 non ATPase-1,2 subunits of the proteasome complex was increased in cells treated with cobalt when compared to vehicle-treated cells (Table 1). Therefore, we investigated whether the proteasome-dependent proteolytic pathway plays a role in the effect of hypoxic stress on AQP5 expression. Addition of the proteasome inhibitor, proteasome inhibitor III (PI III) completely abolished the cobalt induced decrease in expression of AQP5 protein (Fig. 8A, B and D). A second proteasome inhibitor lactacystin (LC), like PI III, also blocked the action of cobalt on AQP5 protein (Fig. 8C and D). Neither PI III nor LC affected AQP5 expression when added alone (Fig. 8 B–G).

**Table 1:** Microarray analysis of cells treated with cobalt vs. vehicle.

Gene symbol	Gene Name	Fold change	p-value	False discovery rate (FDR)
Aqp5	Aquaporin 5	−2.57	6.29E−06	0.0003
Psma5	Proteasome (prosome, macropain) subunit, alpha type 5	+2.41	7.79E−06	0.0003
Psma7	Proteasome (prosome, macropain) subunit, alpha type 7	+2.7	3.58E−06	0.0002
Psmb4	Proteasome (prosome, macropain) subunit, beta type 4	+1.83	7.38E−05	0.0010
Psmb10	Proteasome (prosome, macropain) subunit, beta type 10	+3.24	5.71E−06	0.0003
Psmc1	Proteasome (prosome, macropain) 26, ATPase 1	+2.76	1.86E−05	0.0005
Psmd1	Proteasome (prosome, macropain) 26, non ATPase, 1	+1.95	3.59E−05	0.0007
Psmd2	Proteasome (prosome, macropain) 26, non ATPase, 2	+1.96	4.66E−05	0.0007

Expression of Proteasome subunit encoding genes, that were significantly changed in the presence of hypoxic stress induced by Co^++^. Cells were treated either with vehicle or Co^++^ (n = 3 independent biological samples, n = 3 microarray chips per biological sample). Microarray analysis was performed to identify differentially expressed genes after addition of Co^++^, using R statistical software and the *limma* Bioconductor package.

The Northern blots shown in Fig. 8E and 8F indicate that pre-treatment with PI III or lactacystin inhibited the cobalt mediated decrease in AQP5 mRNA (Fig. 8G), suggesting that hypoxic stress regulates AQP5 transcription via a proteasome dependent mechanism. PI III alone stabilized the expression of HIF-1α (Fig. 8H, lane 4) under normoxic conditions similar to hypoxia (Fig. 8H lane 2) and cobalt (Fig. 8H lane 3). Expression of HIF-1α was not significantly different when PI III was added to cells exposed to hypoxia (Fig. 8H lane 5) and cobalt (Fig. 8H lane 6).

## Discussion

The mammalian alveolar epithelium is anatomically situated at an air-liquid interface, and plays a unique role in both gas exchange as well as fluidization of the lung. Disorders in the regulation of gas exchange or fluid transport are implicated in a legion of pathological outcomes, ranging from the common (such as hypoxia induced lung edema) [8], [9], [21], [22], [23], [24] to the rare (transient tachypnea of the newborn) [25]. Although extensive research has focused on the effects of hypoxia on alveolar epithelial cell function and the mechanisms which impair the ability to efficiently transport fluid across the alveolar epithelium [26], [27], the molecular mechanisms that underlie the physiological processes have only recently begun to be understood. Several reports show that hypoxia induces down-regulation of sodium channel expression and activity in rat lungs and in cultured lung epithelial cells [28], [29], [30], [31], [32]. The decreased function of the sodium channel may be partially responsible for the formation of lung edema under hypoxic conditions. In contrast the effect of hypoxia on the membrane water channel AQP5, which is the major water channel in the alveolar epithelial cells, remains unknown.

We therefore investigated whether AQP5 would respond to hypoxic stress. In this paper we show that AQP5 expression is substantially decreased not only in lungs of mouse exposed to hypoxia but also in a mouse lung epithelial cell line. Both hypoxia as well as inducers of hypoxic stress such as Co^++^ showed similar effects by decreasing expression of AQP5 via a network of pathway(s) that require ERK, HIF-1α and proteasome-dependent degradation.

### Exposure to Cobalt Results in Decreased AQP5 Expression

Exposure to hypoxia (1% O_2_) for 24 h and Co^++^ decreased the expression of AQP5 protein [hypoxia-60%; Co^++^−90%] and AQP5 mRNA [hypoxia-40%; Co^++^−60%] in MLE-12 cells (Figs. 1 and 3). AQP5 expression also decreased in the lungs of mice exposed to hypoxia (Fig. 2) indicating that the results observed in MLE-12 cells are physiologically relevant *in vivo*. We therefore used this cell-line to dissect the molecular mechanisms that lead to decreased AQP5 expression during hypoxic stress. HIF-1α is a well-studied biomarker of cellular hypoxic stress [10], [11], [12], [33]; therefore, its induction was first studied to ensure that MLE-12 cells were under hypoxic stress when exposed to low oxygen concentration (Fig. 1E) or cobalt (Fig. 3E). These results, in lung cells, corroborate other studies which found that hypoxia results in decreased AQP5 mRNA and protein levels in primary cultures of rat astrocytes [34], [35] as well as in fibrotic lung which causes systemic hypoxia [36], and suggest a potential role of AQP5 in the adaptive response to hypoxic stress in alveolar epithelial cells. The relevance of our findings in human pathological conditions such as lung fibrosis may be of clinical interest: Recently, Gabazza et al. [36] showed that AQP-5 expression is decreased in the fibrotic lung of mice treated with bleomycin, and since lung fibrosis often leads to systemic hypoxia, it is possible that it is hypoxia, rather than the fibrosis itself, that results in reduction of AQP5 expression. In contrast, hypoxic stress have been shown to increase expression of another aquaporin isoform, AQP1 in mouse lung and brain [37], in prostate cancer cell line [38] suggesting the existence of complex tissue specific and isoform specific regulatory circuits for the regulation of aquaporins.

### Cobalt Decreases AQP5 Expression by Downregulating Transcription of AQP5 Gene

Decreased levels of AQP5 mRNA could either be due to a decrease in stability of mRNA or decreased transcription of the AQP5 gene. Cobalt did not significantly alter the stability of AQP5 mRNA indicating that the effect is likely at the transcriptional level. To directly test this possibility, we measured hnRNA levels, using real time PCR to quantitatively measure transcriptional levels in hypoxic and non-hypoxic conditions, and found a two-fold decrease in AQP5 transcription after hypoxic stress (Fig. 4B and C). Other investigators have reported that hypoxia regulates the expression of genes at the transcriptional level (such as erythropoietin (EPO), [39], [40] and heme-oxygenase-1 [41], [42]. The lack of significant difference between the hypoxic stress induced decrease in AQP5 protein compared to mRNA levels (Fig. 1 and Fig. 3) indicates that the effect of hypoxic stress on AQP5 is mainly at the transcriptional level. Furthermore, *de novo* protein synthesis is required for this process because addition of the protein synthesis inhibitor CHX not only blocked the effect on AQP5 mRNA but also on AQP5 protein (Fig. 5 A–D).

### HIF-1α is Necessary to Regulate Expression of AQP5 Under Hypoxic Conditions

Because HIF-1α is a “master regulator” of many adaptive responses to hypoxia [43], we investigated whether HIF-1α mediates the effect of hypoxia and hypoxia mimetics on AQP5 expression. Addition of the prolyl hydroxylase (PHD) inhibitor, PHD-0041, stabilizes HIF-1α under normoxic conditions by preventing hydroxylation of HIF-1α, thereby inhibiting proteasome-mediated degradation of HIF-1α [44]. PHD-0041 resulted in decreased expression of AQP5 in a dose-dependent manner similar to the decrease under hypoxic conditions (Fig. 6A, bottom panel). Thus, as the concentration of the PHD inhibitor was increased, PHD enzyme activity decreased, and levels of HIF-1α increased (Fig. 6A top panel), while levels of AQP5 decreased showing an inverse correlation between HIF-1α and AQP5 expression. To test the hypothesis that blocking the activity of HIF-1α would “rescue” the effect of cobalt, we treated MLE12 cells with zinc chloride, which generates an endogenous, dominant negative HIF-1α [20], and in separate experiments, transfected MLE-12 cells with a plasmid encoding dominant negative HIF-1α (HIF-1α with a truncated transcription activation domain in the C-terminal [45], [46]). Figures 6B, 6C, 6E and 6F show that both these treatments “rescue” AQP5 protein. To confirm this, we used the siRNA to knock down HIF-1α as shown in Fig. 6G and 6H and then probed the same blot to determine whether AQP5 protein was “rescued”. Fig. 6I and 6J show that in accordance with the 50% decrease in HIF-1α expression there is a 50% rescue in AQP5 expression. These results, using three independent approaches to test the hypothesis that HIF-1α is critical for transducing the hypoxia mimetic signal to AQP5 unequivocally show that HIF-1α is central to the regulation of AQP5. The role of HIF-1α in response to cobalt appears to be opposite to the role reported for HIF1α in response to osmolar stress [47], where increased osmolality induced an increase in AQP5 expression [47]. While at first glance the seemingly different actions of osmolar stress and hypoxic stress may seem paradoxical, these perturbations are very different in two important ways (1) Hypertonic stress is an acute stressor (acts in seconds/minutes) and is therefore significantly different perturbation than treatment with cobalt (acts in hours/days) (2) The studies by Zhou et al. used transient transfection of AQP5 plasmids, and it is likely that chromatin organization and transcriptional regulation of the transfected episomes is quite different from that of AQP5 in chromosomally associated chromatin. Episomal transient transfection is a very useful tool for studying the “basal transcriptional complex”, but it is a less effective tool when studying the “tissue specific transcriptional complex” as it is less able to pick up the action of long range enhancers and locus control regions.

To determine the signaling pathways which result in downregulation of AQP5 we tested the ERK signaling pathway, which is activated by hypoxic stress [48], [49].The ERK pathway inhibitor (PD98059) blocked the action of hypoxia mimetic cobalt (Fig_._ 7A and B) indicating that in MLE-12 cells cobalt regulates AQP5 expression specifically through ERK signaling pathway. Activation of ERK by cobalt treatment and its inhibition by PD98059 confirmed that ERK signaling pathway was involved in AQP5 down regulation (Fig. 7C and D). Since HIF-1α regulates AQP5 expression Fig. 6, we tested whether ERK regulates expression of HIF-1α in MLE-12 cells. Figs. 6E and F show that HIF-1α is also regulated by the ERK pathway in MLE-12 cells consistent with studies in other cells [50], indicating ERK is upstream of HIF-1α.

### Cobalt Regulates Expression of AQP5 through a Proteasome Dependent Pathway

To identify other elements, which in addition to HIF-1α may regulate AQP5 expression under hypoxic stress; we performed microarray analysis of vehicle-and-cobalt treated cells. This study revealed increased expression of proteasome subunits (Table 1), prompting us to investigate whether expression of AQP5 protein and mRNA under hypoxic stress could be regulated by a proteasome dependent mechanism. Two proteasome inhibitors, proteasome inhibitor III (PI III) and lactacystin (LC) were used to determine the role of the proteasome. PI III is a reversible proteasome inhibitor that reduces degradation of ubiquitin conjugated proteins by the 26S proteasome and displays much higher potency compared to other inhibitors [51]. LC is an irreversible proteasome inhibitor and acts as a highly specific inhibitor of the 20S proteasome (26S proteasome subcomplex, responsible for the actual protease activity) [52], [53], [54].

Proteasome inhibitors completely rescued the effect of cobalt at the mRNA and protein levels, confirming that AQP5 is downregulated by a proteasome-dependent mechanism (Fig. 8). In addition, similar to previous studies [55], proteasome inhibitors also stabilized HIF-1α (Fig. 8H), which is necessary to downregulate AQP5 expression (Fig. 7). Rescue of AQP5 expression by the proteasome inhibitor in the presence of HIF-1α indicates that the proteasome-dependent step is downstream of HIF-1α and that HIF-1α indirectly regulates AQP5 expression in the presence of hypoxic stress via the proteasome dependent mechanism. Rescue of AQP5 mRNA expression by proteasome inhibitors (Fig. 8 E–G) suggests that they are regulating a transcription factor. Many critical transcriptional factors are maintained at appropriate levels by targeted ubiquitination and degradation via the 26S proteasome (complete complex) [54], [56]. It is possible that the proteasome dependent mechanism transduces the hypoxic stress signal to AQP5 by degradation of an inhibitor(s) of its transcriptional repressors, thus resulting in decreased transcription of AQP5. We have ruled out the possibility of degradation of a transcriptional activator because there was no significant increase in AQP5 mRNA levels in presence of proteasome inhibitors. Based on these observations, we it would appear that the regulatory process of AQP5 by HIF-1α described here is somewhat unexpected. Normally in a cell Hif1α is constantly hydroxylated and degraded by the proteasome complex, but in hypoxia the prolyl-hydroxylases are non-functional, and Hif1α induces transcription of its target genes. Our data suggest that an alternative or modified mechanism may exist - proteasome degradation of an inhibitor of the repressor that controls AQP5 transcription. While this may appear to be a complex mechanism, which involves indirect regulation of a transcriptional repressor by HIF-1α, there are known precedents. For example, VHL dependent E-cadherin regulation by HIF-1α is carried out via this indirect form of regulation in clear cell renal carcinoma cells (CC-RCC). Investigators [57], [58] have found that in absence of VHL, HIF-1α is activated and results in decrease in transcription of E-cadherin by activation of transcriptional repressors S1P1 and Snail. The transcriptional repressor Snail is known to repress E-cadherin transcription through the binding of histone deacetylase and promoting repressive changes in chromatin structure [59]. Evans et al [57] also propose that HIF-1α can activate these transcriptional repressors either by direct binding the HRE elements our through activation of TGF-β (Smad mediated signaling pathway). In addition, Mikhaylova et al [60], also observed VHL triggered expression of LC3C by repression of HIF-1 α, which in turn down regulates LC3C by activation of transcriptional repressors. These studies by Evans et al [57], Esteban et al [58], and Mikhaylova et al [60] indicate that HIF-1 α not only activates gene expression but also activates transcriptional repressors. Regulation of downstream genes by these transcriptional repressors further involves other factors for example HDAC indicating a rather complex mechanism of inhibiting gene expression. This role of HIF-1 α and understanding of these complex mechanisms is an emerging field and we anticipate that advancement in this field will resolve these complex questions.

**Figure 9:**
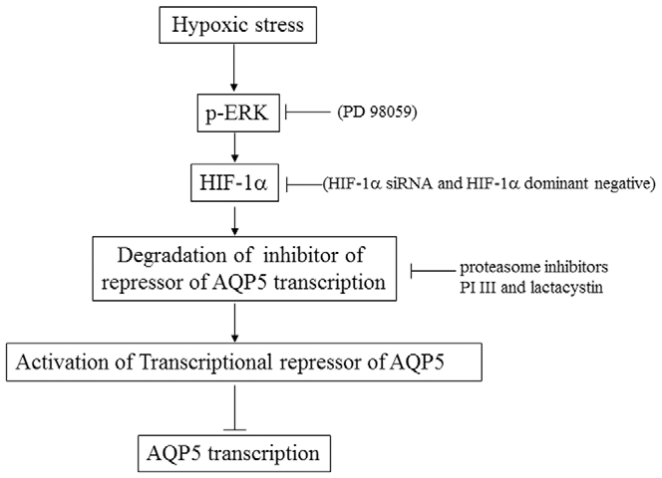
A putative model for the molecular pathway linking hypoxic stress to decreased AQP5 expression. Hypoxic stress activates HIF-1α via the ERK signaling pathway. HIF-1α activates a repressor of AQP5 transcription via a proteasome-mediated mechanism (which could act by degrading inhibitors of the repressor protein).

Based on our results and the findings published in literature we propose a model that outlines the key molecular components of the pathway that transduces the cobalt induced signal to AQP5 (Fig. 9). Hypoxic conditions result in the activation of the ERK signaling pathway, which induces expression of HIF-1α. HIF-1α regulates the function of the transcription factor that is regulated by a proteasome dependent mechanism.

Of special interest is the observation that even within the lung, there is considerable variation in the way aquaporins respond to hypoxia depending on the cell types in which they are expressed. For example, Abreu-Rodríguez et al have recently shown that exposure of mice to hypoxia results in overexpression of AQP1 in the lung (also by a HIF1-α dependent mechanism). Why the two water channels, AQP5 (principally located in the type-1 and type-2 cells of the alveolus) and AQP1 (mainly located in lung capillary endothelia) are regulated in opposite directions by hypoxia is currently unknown. What these studies reveal is a complex and nuanced set of signals and signaling pathways that play a key role in the regulation of water channels in the lung and, in a broader sense, in the physiology of the whole animal.

In summary, we have shown that exposure of mice to hypoxia causes a decrease in expression of AQP5. Using lung epithelial cells we report that cobalt treatment significantly decreases AQP5 expression by a complex signaling mechanism that involves ERK, HIF-1α and proteasome components. Our findings outline a complex molecular interaction for regulation of AQP5 under hypoxic conditions.

## Materials and Methods

### Experimental Reagents

Cobalt chloride (anhydrous) was obtained from Sigma, St Louis, MO. Actinomycin-D (ActD), ZnCl_2_ and the MEK inhibitor PD 98059, which inhibits ERK pathway was purchased from Sigma. Proteasome inhibitor III (PI III), lactacystin (LC) and cycloheximide (CHX) were obtained from Calbiochem, San Diego, CA. PHD inhibitor (FG-0041) was a kind gift from Firbrogen Inc, San Francisco, CA. We used the compound FG-0041 for following reasons (1) in a study by Ivan et al [18], they screened multiple PHD inhibitors and found FG-0041 as a model inhibitor. FG-0041 was specific and was able to inhibit hydroxylation at N-terminal transactivation domain as well as at C-terminal transactivation domain resulting in transcriptionally active HIF. (2) We used various concentrations ranging from 25 µM to 200 µM, similar to those reported in the literature by others - Pacary et al (25 µM to 100 µ M) [61] (3) When observed under the light microscope we did not detect discernible cytotoxicity (ragged cell margins, multiple nuclei etc.) in the time course of the experiment. (4) In addition, dimethyloxaloylglycine is not a selective HIF-PHD inhibitor [19]. Cobalt chloride was dissolved in double distilled water (ddH_2_O). PI III, LC and ActD were dissolved in dimethyl sulfoxide (DMSO) and CHX was dissolved in 70% ethanol. For each experiment, we had a parallel control where cells were treated with DMSO alone as shown in each figure and marked “control”. RPMI 1640 medium with L-glutamine, fetal bovine serum and antibiotics-antimycotics were obtained from Gibco/Life Technologies, Carlsbad, CA. PCR master-mix for quantitative real-time PCR was obtained from Clontech (Mountain View, CA) Target plus pool of HIF-1α siRNA and non-targeting siRNA controls were obtained from Dharmacon (Lafayette, CO).The antibody to β-actin was an anti-mouse monoclonal antibody purchased from Sigma. Anti-HIF-1α monoclonal antibody was obtained from Novus Biologicals, Littleton, CO. Lamin B, ERK and phosphorylated ERK antibody were obtained from Santa Cruz Biotechnology, CA. AQP5 polyclonal antibody (LL639, Lofstrand Laboratories, Gaithersburg, MD) was generated against a synthetic peptide corresponding to the mouse AQP5 carboxyl terminus, and affinity purified on a SulfoLink column (Pierce, Rockford, IL) conjugated with the immunizing peptide [62]. Horseradish peroxidase-conjugated secondary antibodies were from Vector Laboratories (Burlingame, CA). The protein concentration in each sample was measured using the Pierce BCA protein assay reagent kit (Pierce, Rockford, IL). Nitrocellulose membrane, 4–15% linear gradient and 7.5% resolving Tris-HCl ready gels were purchased from Bio-Rad. Immunoreactivity was detected by SuperSignal West Pico chemiluminescent reagents (Pierce).

### Cell Culture

MLE-12 cells were a gift from Drs. Cindy Bachurski and Jeffrey Whitsett (Children’s Hospital Medical Center, Cincinnati, OH) [63]. The growth medium for MLE-12 cells consisted of RPMI 1640 (with L-glutamine) supplemented with 3% fetal bovine serum and 1% antibiotics-antimycotics. Cells were propagated in above growth medium at 37°C with 5% CO_2._

### Hypoxia and Cobalt Treatment

Approximately 48 h before the exposure to hypoxia or cobalt, cells were seeded into six-well tissue culture plates. With the exception of the hnRNA experiments (for which cells were grown in 150 mm plates), all other experiments were performed in six-well plates. Cells were serum starved for 24 h by replacing the medium with RPMI 1640 containing 1% fetal bovine serum as described by Yang *et al.* [64]. Subsequently, the culture medium was replaced by normal growth medium and cells either exposed to different concentrations of cobalt for 24 h or exposed to hypoxia (1%O_2_, 5%CO_2_) in an O_2_-regulated tissue culture incubator (Forma Scientific) for 24 h as described previously [65]. Proteasome, ERK pathway, PHD inhibitor (FG-0041) and transcriptional inhibitors were added in the specified concentrations, 30 min before the cells were exposed to hypoxia or cobalt. For each experiment, we had a parallel control where cells were treated with the solvent used to dissolve the inhibitor (for example DMSO alone for FG-0041) as shown in each figure and were marked “control”. Each experiment was repeated in its entirety at least twice in either duplicate or triplicate.

### Exposure of Mice to Hypoxia

Mice were exposed to hypoxia as previously described [66], [67]. Briefly, mice were randomly assigned to three experimental groups (*n*≥5/group). Sustained hypoxia (8% O_2_) was achieved by housing mice in identical commercially designed chambers (30′′×20′′×20′′) that accommodate up to 24 mice each, and operate under a 12 hour light-dark cycle (Oxycycler model A44XO, Biospherix, Redfield, NY) for 3 and 7 days similar to previous studies [37], [67]. Gas was circulated around each of the chambers, attached tubing and other units at 60 L.min^−1^ (i.e., one complete change per 10 sec). The O_2_ concentration was continuously measured by an O_2_ analyzer, and was maintained throughout the 24 hour circadian cycle by a computerized system controlling the gas valve outlets, such that the moment-to-moment desired oxygen concentration of the chamber was programmed, and adjusted automatically to maintain at a level of 8% O_2_. Deviations from the desired concentration were met by addition of N_2_ or room air through solenoid valves. Ambient CO_2_ in the chamber were periodically monitored and maintained at 0.03% by circulating the gas through soda lime. The gas was also circulated through a molecular sieve (Type 3A, Fisons, UK) so as to remove ammonia. Humidity was measured and maintained at 40–50% by circulating the gas through a freezer and silica gel and ambient temperature is kept at 22–24°C. The experimental protocols were approved by the Institutional Animal Use and Care Committee at the University of Louisville and are in agreement with the National Institutes of Health guide for the care and use of laboratory animals.

### Actinomycin-D Treatment

To determine the stability of AQP5 RNA with or without cobalt, MLE-12 cells were treated for 24 h with 100 µM cobalt and then 10 µg/ml ActD. Cells were collected and total RNA was isolated at the indicated time points.

### Total Protein Preparation and Protein Assay

Following treatment of cells in the specified conditions for the indicated amount of time, the medium was aspirated and the cells were washed with ice-cold phosphate-buffered saline (PBS). Total protein was extracted using Laemmeli buffer without dye (50 mM Tris pH 6.8, 2% SDS, 10% glycerol) plus protease inhibitors. Total protein concentration was determined using Pierce BCA protein assay reagent kit according to manufacturer’s protocol, using bovine serum albumin as the standard.

### Nuclear Extract Preparation

Cells were seeded in 150 mm dishes for 24 h, and then starved for 24 h in 1% FBS culture medium before cobalt (100 µM) treatment or treatment with vehicle alone. After treatment for 24 h, cells were washed with ice-cold PBS, scraped off, and centrifuged for 3 min at 900×g. To 1 volume of pellet, 3 volumes of lysis buffer (10 mM Tris, pH 7.5, 10 mM NaCl, 3 mM MgCl_2_ 0.5% Triton-100) was added, and immediately spun down (4°C, 900×g, 3 min). After removing the supernatant, 0.5 volume of low salt buffer (20 mM HEPES, pH 7.9, 1 mM EDTA, 20 mM NaCl, 20% glycerol) was added and vortexed, followed by slow addition of high salt buffer on ice with agitation (20 mM HEPES, pH 7.9, 1 M NaCl, 1 mM EDTA, 20% glycerol) equal to the volume of low salt buffer. This mixture was then rotated at 4°C for 1 h. and subsequently centrifuged at 13,000 g for 15 min.

### Western Blot Analysis

10 μg of total protein and 20 µg nuclear extract were resolved using SDS-polyacrylamide gel electrophoresis, transferred onto nitrocellulose membrane. The membrane was blocked with 10% milk/TBST (Tris-buffer Saline with Tween 20 [0.1%]) and probed with primary antibodies in 5% milk/TBST overnight at 4°C, followed by 3-quick and then 3×10 min TBST washes, followed by secondary antibody conjugated to Horse Radish Peroxidase enzyme (Vector laboratories) in 5% milk/TBST. The blots were again subjected to 3-quick and then three-10 min TBST washes. Immunoreactivity was detected by SuperSignal West Pico chemiluminescent reagents. Quantitation of the western blots was performed using ImageQuant software (Molecular Dynamics, Sunnyvale, CA). β-actin was used as loading control for total protein and Lamin B was used as loading control for nuclear extract. Protein values are reported as a percentage of protein/loading control when compared with control (100%).

### RNA Isolation and Northern Blot Analysis

Following the specific treatment, total RNA was isolated by using TriReagent solution (Molecular Research Center, Inc., Cincinnati, OH) according to the manufacturer’s instructions. RNA was solubilized in Formazol (Molecular Research Center, Inc.) and RNA concentrations were determined by spectrophotometer and confirmed by agarose gel electrophoresis. 10 μg total RNA of each sample was size-fractionated in a 1% formaldehyde agarose gel and transferred to Hybond N^+^ nylon membrane followed by hybridization and washing as described by Yang et al. [64]. After probing with AQP5, the membranes were stripped in boiling 0.5% SDS for 30 min and were re-probed for expression of L32, a ribosomal protein gene product, as a loading control. Mouse AQP5 and L32 probes were prepared as described previously [64]. Northern blots probed with either AQP5 or L32 were quantified by exposure of a phosphor screen, scanned by means of a Storm 840 scanner, and analyzed using ImageQuant software (all from Molecular Dynamics, Sunnyvale, CA). mRNA values are reported as a percentage of AQP5/L32 compared with the mean values in controls (100%).

### Real Time PCR Analysis

Following exposure to hypoxia or Co^++^ total RNA was isolated by using TriReagent solution (Molecular Research Center, Inc., Cincinnati, OH) according to the manufacturer’s instructions. Total RNA was treated with DNAse (Qiagen) and purified using RNAeasy column (Qiagen) to remove any DNA contamination and this purified RNA was used as a template for cDNA synthesis using cDNA synthesis kit (Invitrogen).

AQP5 and βactin primers were designed to span an exonic and intronic region, so that only hnRNA would be amplified by PCR. The primer sequence used for real-time PCR are listed as follows: AQP5 forward-1-5′-ATCTCTCTGCTCCGAGCCATCTTCTA-3′; AQP5 reverse-1-5′-GAGCATGGAAGGTCT GGTCTGAAA-3′; AQP5 forward-2-5′-AC TTGACTTCCAAGAGCTCAGGCT-3′; AQP5 reverse-2-5′-CTTGCCTGGTGTTGTGTTGTTGCT-3′; β-actin-forward-1-5′GCCTGTACACTGACTTGAGACCAA-3′; β-actin-reverse-1-5′-TGTAGCCCTCCCACTAGATACCAT-3′; β-actin-forward-2-5′-CAGAGAGCTCACCATTCACCATCT-3′; β-actin-reverse-2-5′-ACTCCTGCTTGCTGATCCACATCT-3′. For real-time PCR, 25 µl PCR consisted of 400 pmol of primers, 0.5X of 50,000X stock of SYBR Green I (Molecular Probes, Eugene, OR), 1 unit of Taq polymerase, PCR mix (Clontech) and nuclease-free deionized water and was amplified in DNA Engine Opticon 2 (MJ Research Incorporated). All amplification protocols used a 3 min melting step of 95°C followed by 40 cycles of amplification. Each cycle for various primer sets used an initial melt of 95°C for 15 s, an annealing step of 30 s at optimized temperature, followed by extension step of 60 s at an appropriate temperature, followed by fluorescence measurement. Presence of single peak was considered as a single product, which was verified by running the reactions on agarose gels. Threshold cycle (Ct) values were determined automatically using the Biorad Opticon v3.1 software (Biorad). These values were used to calculate relative expression of AQP5 relative to βactin expression using the ΔΔCt method [68].

### Transfection

cDNA encoding the dominant negative HIF-1α in the pcDNA 3.1(+) (Invitrogen) vector was transfected in MLE-12 cells using Lipofectamine transfection reagent (Invitrogen) according to the manufacturer’s protocol. As a control, empty pcDNA 3.1(+) was similarly transfected in MLE-12 cells. Colonies resistant to 600 µg/mL G418 (Sigma) were isolated. Cells were transfected with HIF-1α and non-targeting siRNA using calcium phosphate as described previously [69]. Briefly, siRNA was precipitated with calcium phosphate in Bes buffered saline (50 mM BES, 280 mM NaCl, 1.5 mM Na_2_HPO4). Cells were incubated with this mixture for 6 hrs at 3% CO_2_. After incubation, cells were treated with fresh media and incubated at 5% CO_2_. Cells were transfected 3 times to achieve knock down of HIF-1α.

### Microarray Hybridization

The Qiagen-Operon (Alameda, CA) mouse 70-mer oligonucleotide library, version 1.1, representing 13,433 known genes, was suspended in 3× standard saline citrate (SSC) and printed at 22°C and 65% relative humidity on aminosilane-coated UltraGAPS slides (Corning; Acton, MA) using a high-speed robotic OmniGrid machine (GeneMachines; San Carlos, CA) with Stealth SMP3 pins from Telechem (Sunnyvale, CA) mounted on a 48-pin head. Spot volumes were 0.5 nL and spot diameters were 75–85 µm. The oligonucleotides were crosslinked to the slide substrate by exposure to 600 mJ of ultraviolet light. For hybridization, the microarrayed oligonucleotides were placed in a hybridization station (Genomic Solutions; Ann Arbor, MI) using Microarray Hybridization Buffer #1 from Ambion (Austin, TX). The hybridization protocol (20) followed a temperature step-down procedure of 65°C, 60°C, and 55°C for 2 h each and 49°C for 10 h (overnight). The slides were washed for 4 min in 0.1× SSC and 0.2% SDS at room temperature with agitation, and four times for 2 min each in 0.1× SSC at room temperature with agitation. The slides were spun dried immediately after washing. More details of slide preparation can be found at http://microarray.uc.edu. Fluorescence-labeled cDNAs were synthesized from 20 µg of total RNA using an indirect amino allyl labeling method (21). The cDNA was synthesized by an oligo (dT)-primed, reverse transcriptase reaction, and the cDNA labeled with monofunctional reactive Cytidine-3 and Cytidine-5 dyes (Cy3 and Cy5; Amersham, Piscataway, NJ). Specific details of the labeling protocol may be found at http://microarray.uc.edu. Imaging and data analyses were carried out using GenePix 4000A and GenePix 4000B scanners (Axon Instruments; Union City, CA) and associated software. The microarray slides were scanned with dual lasers with wavelength frequencies to excite Cy3 and Cy5 fluorescent emission. Images were captured in JPEG and TIFF files, and DNA spots captured by the adaptive circle segmentation method. Information extraction for a given spot was based on the median value for the signal pixels minus the median value for the background pixels to produce a gene set data file for all of the DNA spots. Fluorescence signal intensities of Cy3 and Cy5 were normalized as described later. MIAME standard compliant microarray data have been deposited in the European Bioinformatics Institute database (http://www.ebi.ac.uk/miam express), where it may be retrieved using <MEXP-23> as a query.

### Microarray Data Normalization and Analysis

The data were analyzed to identify differentially expressed genes between cobalt and vehicle treated cells. Three biological replicate arrays, including one dye-flip, were performed. Analysis was performed using R statistical software and the *limma* Bioconductor package [70]. Data normalization was performed in two steps for each microarray separately [71], [72], [73]. First, background adjusted intensities were log-transformed and the differences (M) and averages (A) of log-transformed values were calculated as M = log_2_(X1)−log_2_(X2) and A = [log_2_(X1)+log_2_(X2)]/2, where X1 and X2 denote the Cy5 and Cy3 intensities, respectively. Second, normalization was performed by fitting the array-specific local regression model of M as a function of A. Normalized log-intensities for the two channels were then calculated by adding half of the normalized ratio to A for the Cy5 channel and subtracting half of the normalized ratio from A for the Cy3 channel. The statistical analysis was performed for each gene separately by fitting the following Analysis of Variance model: Y_ijk_ = μ+A_i_+S_j_+C_k_+ ε_ijk_, where Y_ijk_ corresponds to the normalized log-intensity on the i^th^ array, with the j^th^ genotype, and labeled with the k^th^ dye (k = 1 for Cy5, and 2 for Cy3). μ is the overall mean log-intensity, A_i_ is the effect of the i^th^ array, S_j_ is the effect of the j^th^ treatment and C_k_ is the gene-specific effect of the k^th^ dye. Estimated fold changes were calculated from the ANOVA models, and resulting t-statistics from each comparison were modified using an intensity-based empirical Bayes method (IBMT) [74]. This method obtains more precise estimates of variance by pooling information across genes and accounting for the dependency of variance on probe intensity level. False Discovery Rates (FDR) were calculated [75], and genes with FDR adjusted *p*-value <0.05 and that had minimum fold change >2 were considered for further evaluation.

### Statistics

Data are presented as Mean ± SEM and differences within two groups were tested with Student’s t-test and difference within three groups was tested with ANOVA. p<0.05 was considered statistically significant.
